# Valine Radiolysis by H^+^, He^+^, N^+^, and S^15+^ MeV Ions

**DOI:** 10.3390/ijms21051893

**Published:** 2020-03-10

**Authors:** Cíntia A. P. da Costa, Gabriel S. Vignoli Muniz, Philippe Boduch, Hermann Rothard, Enio F. da Silveira

**Affiliations:** 1 Physics Department, Pontifícia Universidade Católica do Rio de Janeiro, Rua Marquês de São Vicente 225, Rio de Janeiro 22451-900, Brazil; cintia-apc@hotmail.com (C.A.P.d.C.); enio@vdg.fis.puc-rio.br (E.F.d.S.); 2 Physics Institute, Universidade de São Paulo, Rua do Matão, Sao Paulo 1371–05508-090, Brazil; gabrivignoli@gmail.com; 3 Centre de Recherche sur les Ions; les Matériaux et la Photonique. Normandie Université, ENSICAEN, UNICAEN, CEA, CNRS, CIMAP, 14000 Caen, France; boduch@ganil.fr

**Keywords:** amino acid, valine, MeV ion irradiation, radiolysis, infrared absorption spectroscopy, destruction cross section, stopping power dependence

## Abstract

Radiolysis of biomolecules by fast ions has interest in medical applications and astrobiology. The radiolysis of solid D-valine (0.2–2 μm thick) was performed at room temperature by 1.5 MeV H^+^, He^+^, N^+^, and 230 MeV S^15+^ ion beams. The samples were prepared by spraying/dropping valine-water-ethanol solution on ZnSe substrate. Radiolysis was monitored by infrared spectroscopy (FTIR) through the evolution of the intensity of the valine infrared 2900, 1329, 1271, 948, and 716 cm^−1^ bands as a function of projectile fluence. At the end of sample irradiation, residues (tholins) presenting a brownish color are observed. The dependence of the apparent (sputtering + radiolysis) destruction cross section, σ_d_, on the beam stopping power in valine is found to follow the power law σ_d_ = aS_e_^n^, with *n* close to 1. Thus, σ_d_ is approximately proportional to the absorbed dose. Destruction rates due to the main galactic cosmic ray species are calculated, yielding a million year half-life for solid valine in space. Data obtained in this work aim a better understanding on the radioresistance of complex organic molecules and formation of radioproducts.

## 1. Introduction

Amino acids are building-blocks of proteins, essential in the dynamics of all living organisms. Moreover, they also play an important role in cell signalization, and in the innate immune system. These stable organic molecules are constituted by carbon, hydrogen, oxygen, and nitrogen. Sulfur is also present in some species. The amino acids molecular structure is constructed by one central carbon atom, called α-carbon, linked to four groups: the carboxyl radical, –COOH, the amino radical, –NH_2_, a hydrogen atom, –H, and a side chain, –R. Two important chemical properties are caused by the interaction between the carboxyl and amino radicals. The first one is related to the dipole moment of the amino acid, which is different in solid and gas phases. Indeed, in solid phase, the mentioned structure is preserved (particularly at high temperatures). For both polar and apolar side chains, the molecule has a significant dipole moment and is called zwitterion. In gas phase, the dipole moment is weak and the molecule is called “neutral”. In condensed phase, the proton of the –COOH group migrates to the amino one forming the zwitterion. The second chemical property concerns to the peptide bond which occurs when the hydroxyl radical of the COOH group is induced to react with the NH_2_ radical attached to another amino acid molecule, delivering a H_2_O molecule and synthesizing a peptide through a bond between the CO and NH radicals. Endoergic reactions may occur several times with nearby amino acids producing larger peptides [1].

The discovery of amino acids in meteorites [2,3], in comets [4], and possibly in the interstellar medium [5,6,7,8] inscribed them into the portfolio of astrophysical materials. Nevertheless, finding the origin of amino acids (as well as any prebiotic material, in general) is a central question in Astrobiology. The asteroids and comets that heavily bombarded the Earth circa 4 billion years ago [9,10] probably transported prebiotics. How relevant were these molecular species for triggering life on Earth? Furthermore, given the omnipresent radiation in outer space and around the giant planets [11], once formed in astrophysical environment, can amino acids survive the interplanetary journey? How could they survive the harsh conditions of the early Earth?

Ionizing radiation processes are largely used in industry, in the production and/or to increase the lifetime of biological materials, e.g., ionizing radiation induces genetic modifications in plant seeds. Therefore, knowledge about the radioresistance of complex organic molecules and the subsequent formation of radioproducts may also have importance in medical science and in the industry, as for instance in food production [12], pharmaceutics [13], and particularly in cancer treatment, shielding astronauts in space, and treating victims of irradiation by nuclear materials. Except for glycine, amino acids can exist in two optical isomeric forms, known as L and D; only L-amino acids form proteins; D-amino acids can be found in antibiotics [14] and in some bacteria cell walls [1,15]. Studies on amino acid radiolysis have already been performed. In particular, for glycine, the simplest amino acid, Gerakines et al. studied thermal effects on its dissociation by energetic protons [16]. Pilling et al. irradiated glycine with 1 MeV H^+^ in order to study the stability of different polymorph configurations [17]. Pilling et al. [18], Maté et al. [19], and Souza-Corrêa et al. [20] studied its dissociation by keV electrons. UV radiolysis was performed by Peeters et al. [21] and by Ferreira-Rodrigues et al. [22]. For valine, electronic sputtering by MeV heavy ions was discussed by Salehpour et al. [23], Becker et al. [24], and Sundqvist et al. [25].

The current work aims at investigating some aspects of amino acid radiolysis. Specifically, the goal is to determine amino acid destruction cross sections by light and heavy energetic projectiles over a large projectile energy range. This huge experimental task is strongly reduced if amino acid radiolysis proceeds similarly as for condensed gases: their destruction cross sections depend only on the electronic stopping power (S_e_) of the ion-target system and not on specific quantities such as kinetic energy, mass, and atomic number of the projectile, as well as molecular and crystalline structures of the target [26,27,28,29,30]. Based on these premises, we have selected the amino acid D-valine (Val) to be irradiated with MeV H^+^, He^+^, N^+^ and 230 MeV S^15+^ ions, the latter for obtaining a much higher S_e_. This compound is hydrophilic, has molar mass 117.15 g·mol^−1^, density 1.32 g·cm^−3^, and chirality properties [31]. Although observed in meteorites [1,2], d-valine does not participate in the known biological processes; l-valine, the chiral form of the d-valine, is the isotopomer existent in living organisms, also occurs in astrophysical environment and – except for the optical activity—has the same characteristics. Fourier transform infrared absorption spectroscopy (FTIR) at room temperature was used to monitor the valine radiolysis.

Data obtained in this work are a contribution for a better understanding on the radioresistance of complex organic molecules, as well as on the formation of radioproducts; our findings may have consequences to Medical Science [32] and to Astrobiology [33]. The work also shows that, although the sample degradation could be adequately monitored by any vibrational band, the absorbance evolutions as a function of fluence exhibit variations for different bands. This finding may be particularly relevant for astrophysical observations.

Irradiations with low electronic stopping power projectiles were performed using 1.5 MeV H^+^, He^+^, and N^+^ ion beams provided by the Van de Graaff Laboratory at the Pontifícia Universidade Católica do Rio de Janeiro (PUC-Rio). Irradiations with 230 MeV S^15+^ projectiles were carried out at GANIL (Grand Accélérateur National d’Ions Lourds), Caen, France.

## 2. Results

When a solid is bombarded by ion beams, at least three relevant material processes occur: (a) structural rearrangement of molecules, (b) induced chemical reactions (rearrangement of atoms) and (c) sputtering (emission of atoms and molecules). All the three processes can be monitored by infrared spectroscopy. Successive acquisition of valine infrared spectra during bombardment with different ion beams show that they are very similar: for this reason, only the FTIR spectra obtained with the H^+^ and S^15+^ beams are displayed. In fact, the major difference observed in the experiments is the absorbance decrease rate: for the same beam flux, the higher the stopping power of the projectile in the sample, the greater is the disappearance rate of valine molecules from the sample. The crystalline rearrangement (crystallization, amorphization, pore collapsing/compaction) is a process observable by FTIR in the very beginning of irradiation and does not involve the dissociation or removal of valine molecules.

### 2.1. Results from the Van de Graaff Laboratory: H^+^, He^+^ and N^+^ Ion Beams

#### 2.1.1. 1.5 MeV H^+^ Beam Irradiation

**Figure 1:**
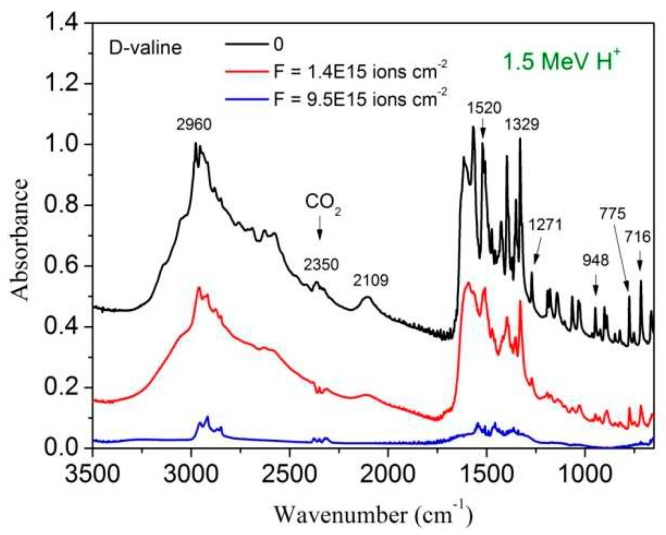
Evolution of the valine infrared spectrum as the projectile fluence increases. The upper spectrum corresponds to the virgin sample; the two others were acquired at fluences 1.4 × 10^15^ and 9.5 × 10^15^ ions cm^−2^, respectively. The feature at ~2350 cm^−1^ is due to atmospheric CO_2_ outside the chamber but inside the FTIR spectrometer; its absorbance varies up and down with time.

Figure 1 shows the FTIR spectrum evolution for three fluences of the 1.5 MeV H^+^ beam: (i) zero fluence or virgin sample (black line, with the highest absorbances); (ii) F = 1.4 × 10^15^ ions cm^−2^, an intermediate fluence (red line, spectrum in the middle); and (iii) F = 9.5 × 10^15^ ions cm^−2^, the highest fluence (blue line, the lowest absorbances). For the highest fluence, all valine molecules of the sample have been degraded: the still existing bands are attributed to daughter molecules (products). The only exception is the double peak at ~2350 cm^−1^ which is due to atmospheric CO_2_ existing outside the chamber but inside the sight of the spectrometer IR beam (its absorbance varies with time, not with fluence).

**Figure 2:**
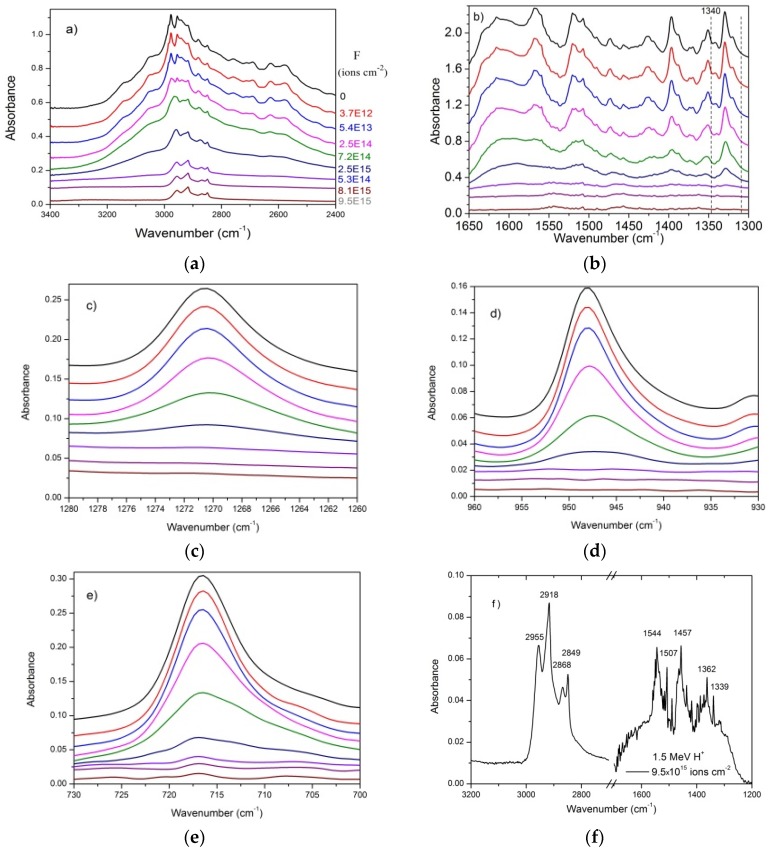
Decrease of valine absorbance as the 1.5 MeV H^+^ beam fluence increases for five selected IR regions (**a**–**e**). Note in particular that absorbances of 1329, 1271 and 948 cm^−1^ bands disappear completely, while those of 2955, 1507 and 716 cm^−1^ bands do not. The fluence legend for Figure 2a is the same for all figures. Panel (**f**) is a zoom of (**a**) and (**b**), for the highest fluence.

In Figure 2, the spectrum evolution with fluence is presented for some selected bands. Data are the same as Figure 1 but, in this case, spectra for nine fluences are shown for: (a) 3400–2400 cm^−1^ and (b) 1650–1300 cm^−1^ ranges, and (c) 1271, (d) 948, and (e) 716 cm^−1^ bands. At first sight, all bands seem to decrease proportionally with fluence; but, after detailed inspection, different behaviors can be remarked specially at the beginning (due to compaction) and at the end (due to the appearance of product bands, Figure 2f) of irradiation. At the end of irradiation the samples (residues) present a brownish color. In reference to the pioneer work of Sagan and Khare [34], these radioproducts (daughter molecules) are referred to as tholins.

The fact that the 1544 and 1457 cm^−1^ features increase along the irradiation (dominating the IR spectrum at the end) indicates that they are not valine bands; instead, they belong to valine daughter molecules. Furthermore, the 3300–2400 and the 1500–1300 cm^−1^ spectral regions, as well as the 716 cm^−1^ band, present a slower decrease rate and do not disappear at the final fluence; they should be formed by two overlapping components, one due to precursor molecule bands, the other due to product bands. Table 1 presents the wavenumber of the main observed valine bands. Here, the third column displays the wavenumber of products after H^+^ irradiation.

**Figure 3:**
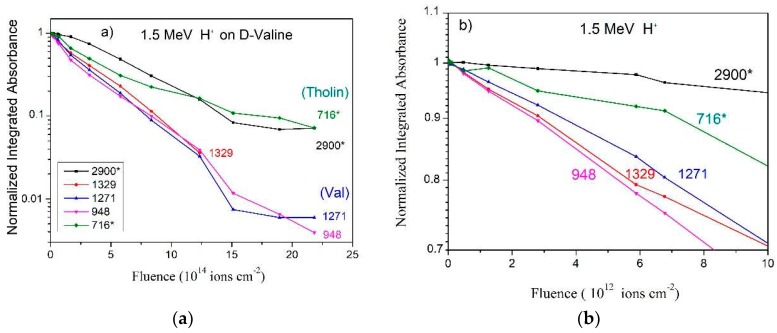
Dependence on fluence of the integrated absorbance for several bands observed in the H^+^ beam irradiation:(**a**) up to the fluence 25 × 10^14^ ions cm^−2^; (**b**) up to the fluence 1 × 10^13^ ions cm^−2^. Data are normalized to 1 at F = 0. The smaller slopes at high fluences exhibited by the 2900* (3300–2400, shown in Figure 2a) and 716* (Figure 2e) cm^−1^ bands are attributed to the rising of product’s bands at very close wavenumbers. (*) means overlapping with product’s band. Colored lines are guides for eyes.

The integrated absorbance evolution with H^+^ fluence is presented in Figure 3 for several valine bands. The absorbance is normalized to 1 at F = 0, in order to compare compaction effects on different bands. Two groups can be recognized: the first one, the “tholin” group [34], is constituted by the 2900 (integrated from 3300 to 2400 cm^−1^) and 716 cm^−1^ bands which present a slow decrease with fluence; another, the “pure valine group”, is constituted by the 1329, 1271 and 948 cm^−1^ bands. The tholin group includes bands of daughter molecules, which absorb IR wavelengths close to the valine bands. Consequently, the bands assigned to this group do not vanish completely, in contrast to the behavior of pure valine group. The absorbance decrease at low fluences is emphasized in Figure 3b. At the very beginning of irradiation, absorbances of the pure valine group exhibit an exponential behavior, while absorbances of the 716 cm^−1^ band and in particular of the 2900 cm^−1^ band are approximately constant with increasing fluence. Our interpretation is that these two bands are sensitive to compaction, while the other three are not. This means that valine porous samples are more transparent than compacted ones in the 3300–2400 cm^−1^ IR region.

#### 2.1.2. 1.5 MeV He^+^ and N^+^ Beam Irradiations

**Figure 4:**
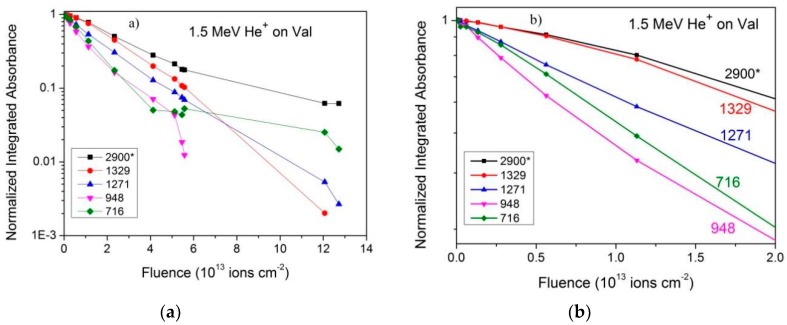
(**a**) Dependence on fluence of the integrated absorbance for several bands observed in the He^+^ beam irradiation. Data are normalized to 1 at F = 0. The asterisk indicates overlapping with product’s bands. (**b**) Zoom into the low fluence region. Colored lines are guides for eyes.

**Figure 5:**
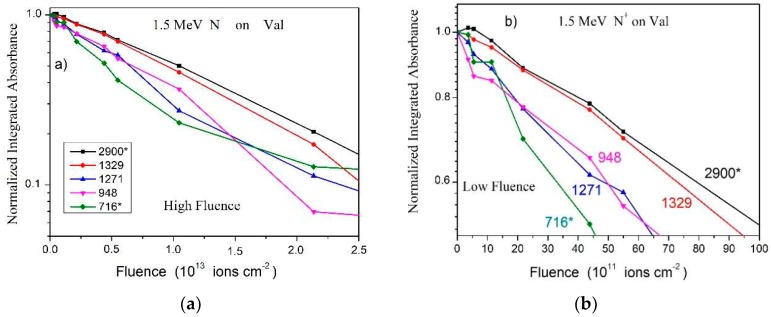
(**a**) Dependence on fluence of the integrated absorbance for several bands observed in the N^+^ beam irradiation. (**b**) Zoom into the low fluence region. Data are normalized to 1 at F = 0. Colored lines are guides for eyes.

Absorbance evolution obtained for the 1.5 MeV He^+^ and N^+^ irradiations are presented in Figure 4 and Figure 5, respectively. Data for the same five bands analyzed for the H^+^ irradiation are again depicted. For the He^+^ beam data, the integrated absorbances are normalized at F = 0, to facilitate a comparison of their slopes. Compaction and product formation (for the 2900 and 716 cm^−1^ bands) are observed for low and high fluences, respectively.

Concerning the N^+^ beam data, Figure 5a shows the absorbance evolution of five valine bands, normalized at F = 0. In agreement with observations for irradiations with other ion beams, the slopes of the 2900 and 716 cm^−1^ bands are lower, especially at high fluences, due to the contribution of product absorbances.

Compaction effects are clearly seen for H^+^ (Figure 3) at the beginning of irradiation; for He^+^ (Figure 4) and N^+^ beams (Figure 5a and its zoom in Figure 5b), this effect is particularly noticeable for the 2900* cm^−1^ band.

### 2.2. Results from GANIL (CIMAP Laboratory)

**Figure 6:**
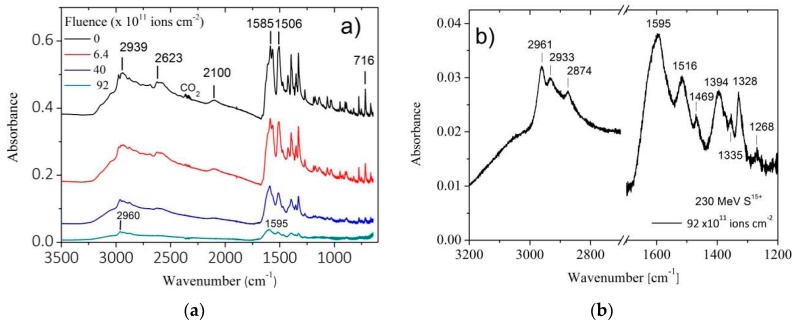
(**a**) Valine IR spectra as a function of projectile fluence. The first spectrum at the top corresponds to the non-irradiated sample and the 4th one was acquired at the end of the irradiation. (**b**) Zoom of the 3200–2700 and 1700–1200 cm^−1^ regions for the highest fluence measurement.

**Figure 7:**
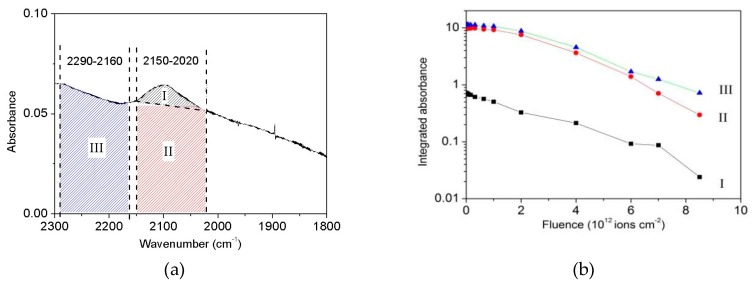
(**a**) Valine IR spectrum around the 2109 cm^−1^ band. The three regions selected correspond to: (I) the 2109 cm^−1^ band, (II) background below this band, and (III) background next to it. (**b**) The integrated absorbance evolution with projectile fluence for these three spectrum regions.

The overall IR spectrum evolution of valine bombardment by 230 MeV S^15+^ projectiles is presented in Figure 6a. As irradiation proceeds, absorbance decreases equally for all bands. This is a hint that their correspondent A-values do not depend strongly on fluence. In other words, a given band does not disappear faster than another, so that the deduced valine destruction cross section is about the same regardless of the band considered. This is true for both narrow and broad bands. The latter include large and complex structures, such as the 3200–2400 cm^−1^ and 1700–1200 cm^−1^ regions: the whole feature belongs to valine and collapses into product bands as fluence increases (Figure 6b, acquired for the highest fluence). As an illustration, Figure 7 compares the decrease of three integrated absorbances with fluence: the (I) region corresponding to the well-defined 2109 cm^−1^ band, the (II) region of the corresponding baseline structure, and the (III) region next to (II) with the same wavenumber width (Figure 7a). At high fluences, all of the three integrated absorbances decrease exponentially and proportionally to each other (Figure 7b). At low fluences, compaction effects are not visible for absorbance of (I) region but are clearly seen for (II) and (III) regions. The sample was irradiated up to 0.92 × 10^13^ ions cm^−2^, corresponding to a dose of 23 eV molecule^−1^. The 1329, 1271 and 948 cm^−1^ bands have disappeared at the end of irradiation, evidence that valine was totally degraded. On the other hand, as presented in Figure 6b for the 3200–2200 and 1700–1200 cm^−1^ regions, several bands are still seen at the end of irradiation, reason why they are attributed to daughter molecules. In Table 1, the fourth column lists the main product’s bands observed at the highest fluence with possible attributions.

**Figure 8:**
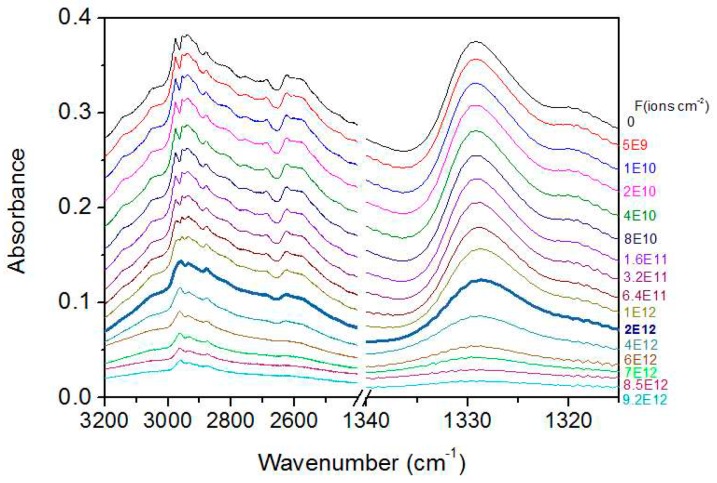
Absorbance decrease for the 3200–2400 and 1340–1315 cm^−1^ regions, as a function of projectile fluence. The first spectrum (top) and the one marked by the thicker line correspond to the non-irradiated sample and to a fluence of 2 × 10^12^ ions cm^−1^, respectively. Note the disappearance of minima at high fluences. The legend at the right side expresses fluence in ions cm^−2^.

Figure 8 depicts the same data as in Figure 6 but zooming into the 3200–2400 and 1340–1315 cm^−1^ regions; intermediate fluences are now included. Note that: (a) no significant peak shifts are observed; (b) peaks become moderately broader at high fluences (the thicker line for fluence ~2 × 10^12^ ions cm^−2^ may be taken as a reference); (c) some peaks (e.g., at 1329 cm^−1^) disappear at high fluences but others (e.g., at 2961 cm^−1^) do not, which provides evidence that these are residues. The peak broadening causes the disappearance of minima between some bands.

**Figure 9:**
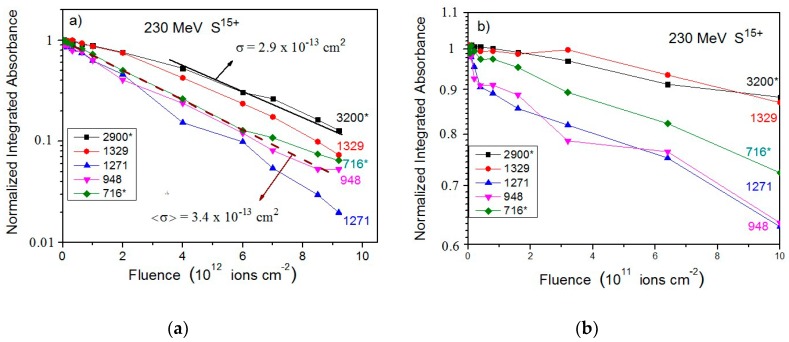
(**a**) Integrated absorbance decrease of five valine bands as a function of 230 MeV S^15+^ projectile fluence. Data have been normalized to unity for the virgin sample. The dashed line represents degradation with the average cross section <σ>. (**b**) Zoom of the same data for the low fluence region. (*) means overlapping with product’s band.

The dependences of the integrated absorbances on fluence for the 3300–2400, 1335–1304, 1279–1261, 957–937, and 726–705 cm^−1^ bands are depicted in Figure 9 for (a) high fluences and (b) low fluences. Clearly, the slopes are close but not identical. Four possible reasons are: (i) compaction, (ii) growth of products, (iii) uncertainty of the baseline selected for the absorbance calculation, and (iv) dependence of A-values on fluence. In detail:

1. Compaction effects have been observed to occur at low fluences and differently for distinct bands. However, since this is a property of the material, the different absorbance evolutions are expected to be similar for any ion beam. Indeed, comparing Figure 3b with Figure 9b, it can be seen that—for both irradiations—the 948 cm^−1^ band absorbance is the one that decreases the most, while those of the 2900 and 1329 cm^−1^ bands decrease much less. The compaction effect modifies absorbances up to 20%.
2. Product bands: For low fluences, the concentration of daughter molecules is still too low for disturbing the chemical environment in such a way to modify precursor’s absorbances (this effect is nevertheless expected to be seen at high fluences). Furthermore, the absorbance slopes do not seem to be correlated with product formation. Figure 8 and Figure 6b show that the 1271, 948, and 716 cm^−1^ bands are due only to valine and, consequently, their absorbances decrease to zero at the end of irradiation; the 3300–2400 and 1700–1300 cm^−1^ (large) regions contain contributions of daughter molecules and their absorbances decrease more slower. The 716 cm^−1^ band is well seen in the non-irradiated valine spectrum, but contrarily to what happens with H^+^ irradiation, this band is not observed at the last fluence (Figure 6a). This may be explained by the fact that thickness of the sample irradiated by the S^15+^ beam was half of that irradiated by the H^+^ beam, preventing small (valine or product) peaks to be observed in the highest fluence spectrum. The large background in the 1700–1200 cm^−1^ region is probably due to amide compounds. Consistently, Figure 9a shows that the 2900 and 716 cm^−1^ bands have a lower slope at high fluences, indicating overlapping with product’s bands.
3. Baseline selection: Figure 7a,b show that the baseline is not critical, since the baseline and peak evolutions are similar. However, once baseline is generated by very large vibrational bands, these may have distinct sensitivities to compaction.
4. Chemical environment changes: Irradiation modifies the crystalline structure (compaction, amorphization and crystallization). Therefore, a moderate dependence of A-value on constituent concentrations is expected. An overview of the A-value variation, before and after compaction, is presented in Table 2 for the four different ion beams.

Inspection of the data presented in Table 2 reveals that:

1. For non-irradiated samples, the dispersion of the relative A-values are about 20%. This is probably due to non-homogeneous samples. Porosities may be different. No particular correlation between the relative A-values with sample thickness is observed.
2. For non-irradiated samples, the relative A-value variations are band-dependent. For the H^+^ beam, the relative A-values for the 2900 and 716 cm^−1^ bands have increased by a factor ~2 from F = 0 to F = F_ref_; these two bands are compaction sensitive (Figure 3b); for the other bands, the relative A-values are close.
3. Relative A-value variations are lower for the N^+^ and S^15+^ beams. A possible explanation is that these beams have larger stopping power than the two others.

This analysis confirms that a procedure to separate compaction effects from radiolysis/sputtering degradation is to analyze absorbance at different fluences. At the beginning of irradiation, effects due to all three processes are observable; after a certain fluence, which depends on the beam stopping power, the compaction process is over. Annealing would be another procedure to promote compaction and inducing crystallization.

### 2.3. Cross Section Measurements

**Figure 10:**
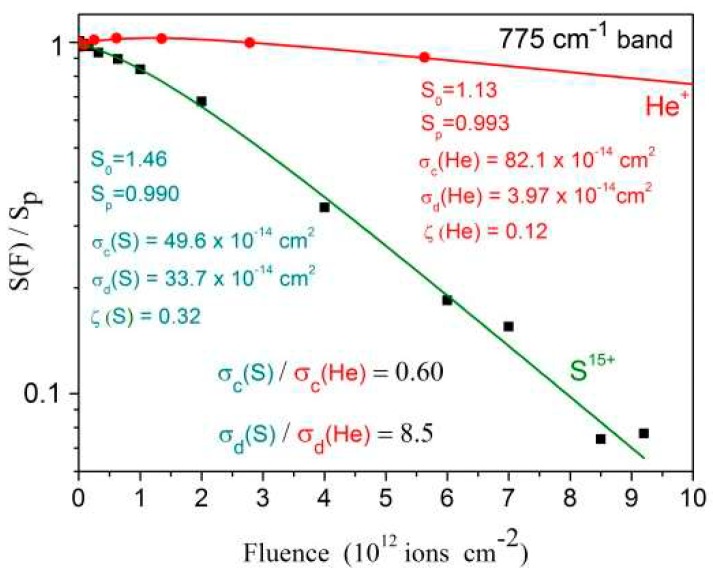
Integrated absorbance decrease for the 775 cm^−1^ band as a function of fluence for the 1.5 MeV He^+^ and the 230 MeV S^15+^ beams. Fittings are performed with Equations (7) and (8) (Section 4.2).

Figure 10 illustrates the concepts presented in Section 4.1 for the S^15+^ and He^+^ ion beam irradiations, using absorbances of the 775 cm^−1^ band. The measured apparent destruction cross sections are quite distinct, σ_d_^ap^(S)/σ_d_^ap^(He) = 8.5, while the compaction ones are close to each other, σ_c_(S)/σ_c_(He) = 0.60. The relative porosities of the two samples used for irradiation with the S^15+^ and He^+^ ion beams are ζ = 0.32 and ζ = 0.12, respectively. Note that, for both irradiations, the sample porosity has the effect to decrease the absorbances at wavenumbers around 775 cm^−1^. Once compaction starts to remove pores at the beginning of irradiation, the absorbance increases even if the real column density decreases due to radiolysis and sputtering. For low fluence He^+^ beam, the compaction effect dominates over those of the other processes, so that S(F)/S_p_ remains greater than 1 up to F ~ 2/σ_c_ = 2.4 × 10^12^ ions cm^−2^. For much higher fluences, the S(F) evolution is dominated by the radiolysis and sputtering.

Table 3 offers an overview of the measured apparent destruction cross sections for the all the ion beams, based on five selected bands; as illustration, one extra band (782–763 cm^−1^) was analyzed for the He^+^ and S^15+^ ion beams to extract σ_c_. The conclusion, already anticipated by the results exhibited in Figure 3, Figure 4 and Figure 5, is that cross sections vary according the selected band. These variations should not be attributed to statistical fluctuations since distinct irradiations yield similar discrepancies. For describing this systematic variation supported by experimental data, the parameter Δσ_j_, defined in Equation (9) (Section 4.2), is introduced.

The 230 MeV S^15+^ beam data were analyzed in the same way. In order to evaluate the influence of the chemical environment on absorbance for each vibration mode, σ_d_^ap^ was determined for the S^15+^ irradiation, based on data plotted in Figure 9a. Results are presented in Table 3. The mean value is σ_d_^ap^ = (33.7 ± 4) × 10^−14^ cm^2^. Data presented in Figure 9a are evidence that absorbance evolutions of bands are not equal to each other but similar, so that the σ_d_^ap^ extracted from them are distinct. Futhermore, since compaction and product formation are not responsible for such discrepancies, the conclusion is that A-values of band j should depend on constituent concentrations. For the S^15+^ irradiation, Δσ_j_ was determined by fitting data with Equation (9) and the obtained values are also shown in Table 3. According to this model, Δσ_j_ should be a characteristic of the band and, therefore, the evolution pattern of the bands should be the same for all ion beams. A comparison of Figure 9a with Figure 3a, Figure 4, and Figure 5a shows that this is indeed the case.

### 2.4. Summary of the Experimental Results

The methodology used to obtain the results of Figure 10 and Table 3 (see Section 4.2) was also applied to the other measurements. The corresponding cross sections and relevant experimental characteristics of the ion beams interacting with valine are summarized in Table 4. Average values of the initial column density, N_0_, and of the thickness, T_k_, are obtained from analysis of the 716 cm^−1^ (A_ν_ = 3.37 × 10^−18^ cm/molec) and the 948 cm^−1^ (A_ν_ = 1.04 × 10^−18^ cm/molec) bands; σ_d_^ap^ is determined by the average behavior of several bands.

## 3. Discussion

As fluence increases, three evident characteristics can be seen in the spectra presented in Figure 1 and Figure 2: (i) the overall spectrum shape remains approximately the same, (ii) the spectral resolution deteriorates, and (iii) there is an exponential decrease of absorbance.

In general spectrum shapes are preserved during irradiation, evidence that the absorbance decrease on fluence is quite uniform over the analyzed spectrum range. Comparing the spectrum of the virgin sample with the one obtained at F = 1.4 × 10^15^ ions/cm^2^ for H^+^ ions, small differences can be recognized: in the irradiated sample spectrum, peaks are larger and peak/baseline ratio is lower. The shoulder at ~3200 cm^−1^ visible in the virgin sample spectrum disappears at F = 1.4 × 10^15^ ions/cm^2^. The same occurs for the 2109 cm^−1^ band, which is clearly seen in the former but is well reduced in the latter. Features visible around 2300 cm^−1^ are due to CO_2_ contamination outside the chamber, but inside the spectrometer. Figure 3, Figure 4, Figure 5 and Figure 9, drawn in a semi-log plot, show that the trend of the integrated absorbance decrease is exponential. Discrepancies from this function are due to compaction (at low doses) and overlapping with daughter molecule bands (mainly at high fluences). With different ion beams, for already compacted samples and for pure precursor bands, slopes for distinct bands are systematically different from each other. Since this fact happens for precursor bands that do not overlap with product ones, it strongly suggests that A-values are not constant during irradiation. We propose that A-values have a weak exponential dependence on fluence, which has to be taken into account due to the exponential decrease of the precursor concentration and, consequently, due to the exponential increase of the concentration of products.

Literature data on amino acid radiolysis by MeV ions are poor. Two similar works on glycine bombarded by MeV H^+^ are available: Gerakines et al., 2012 and Pilling et al., 2013 [16,17]. In the former, for glycine at 140 K irradiated by 0.8 MeV H^+^, destruction cross section was determined to be 0.12 × 10^−14^ cm^2^; it is expected that at 300 K this value be slightly higher. In the latter, for glycine at 300 K irradiated by 1.0 MeV H^+^, the cross sections 0.1 to 0.5 × 10^−14^ cm^2^ were determined for β-Gly and 2 to 3 × 10^−14^ cm^2^ for α-Gly, respectively; cross sections for α-Gly are higher, but they may be affected by compaction, a process better understood nowadays. These results are in good agreement with the values 0.14 to 0.42 × 10^−14^ cm^2^ obtained for valine in the current work for 1.5 MeV H^+^.

### 3.1. Dependence of Cross Sections on the Electronic Stopping Power

**Figure 11:**
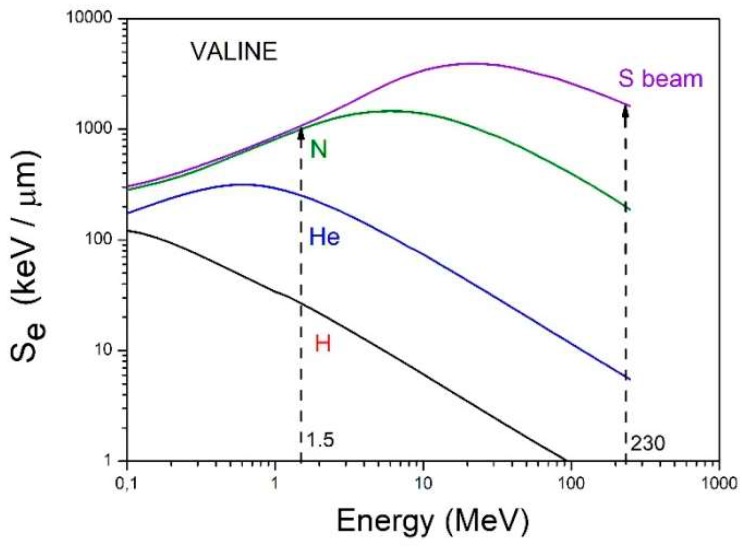
Dependence of electronic stopping power on projectile (H, He, N and S) energy. Dash lines indicate the two ion beam energies used in the current work.

The dissociation of a molecule in a highly excited electronic state is very likely and therefore the dependence of its destruction cross section on the electronic stopping power S_e_ is expected. Figure 11 depicts the evolution of S_e_ with the projectile energy for the four beams used. A pertinent question is whether this dependence is linear or not, revealing if dose is the relevant quantity in degradation by radiolysis. This analysis may be performed dividing absorbance values by S_e_ and comparing the normalized integrated absorbance evolution with fluence for the different ion beams.

**Figure 12:**
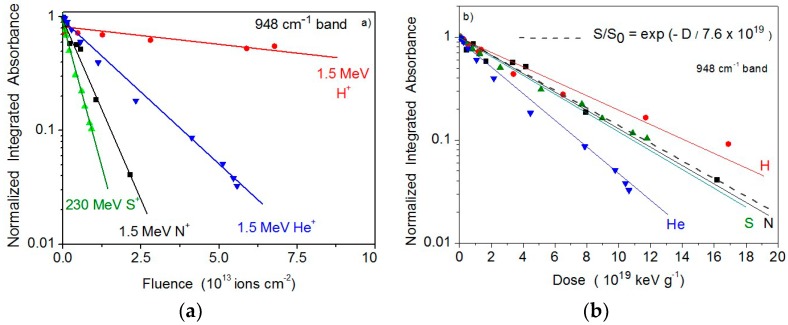
(**a**) Evolution of the 948 cm^−1^ band integrated absorbance on fluence for H, He, N and S ion beams. In semi-log plot, angular coefficients represent cross sections. Integrated absorbances have been normalized to 1 at F = 0. (**b**) Same data, but plotted as a function of the absorbed dose.

Figure 12a shows how the integrated absorbance of the 948 cm^−1^ band varies with fluence for each irradiation; all data are divided by the initial integrated absorbance, S_0_, which means that they are normalized to unity for the virgin sample. The exponential decrease is evident for the four measurements. It is also clear that the higher the stopping power the steeper the absorbance decrease. In order to compensate this effect, absorbance is plotted as a function of absorbed dose, D, defined as the projectile energy, deposited in a given volume of the sample, divided by the mass in that volume: D = S_e_ F/ρ, where ρ is mass density. Figure 12b presents the same data exhibited in Figure 12a but now as a function of D. The span in the evolution due to the different beams is strongly reduced: except for the He beam, data fluctuate around the dashed line, corresponding to the average function S/S_0_ = exp(−σ_d_^ap^ F) = exp(−D/D_mean_), where D_mean_ = S_e_/(ρ σ_d_^ap^) is the mean dose to destroy valine. For instance, for the 1.5 MeV N^+^ beam, <σ_d_^ap^>= (11 ± 4) 10^−14^ cm^2^, D_mean_ = 6.9 × 10^19^ keV/g = 1.1 × 10^7^ Gy.

**Figure 13:**
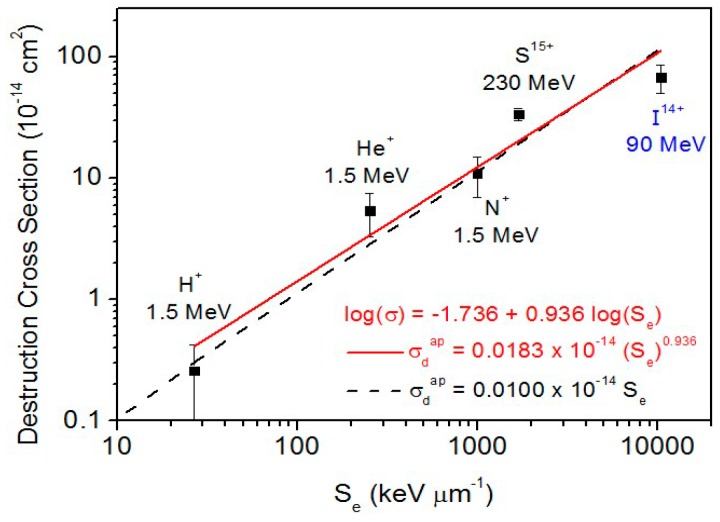
Dependence of the apparent destruction cross section on electronic stopping power. Dash line corresponds to σ_d_^ap^ proportional to S_e_. For the fitting parameters, σ_d_^ap^ and S_e_ are expressed in cm^2^ and keV/μm, respectively. 90 MeV ^127^I projectile data were obtained with secondary ion mass spectrometry.

As discussed before, radiolysis and sputtering are supposed to have the same dependence on fluence and cannot be (easily) determined individually: what is experimentally determined by this methodology is the sum of the two effects, namely the apparent destruction cross section of valine. This quantity, σ_d_^ap^, is displayed in Figure 13. The current measurements indicate that σ_d_^ap^ is approximately proportional to the electronic stopping power:

σ_d_^ap^ = a (S_e_)^n^ (1)

where the best fitting yields a = 1.8 ×10^−20^ cm^2.936^/keV^0.936^ and *n* = 0.936; σ_d_^ap^ and S_e_ are expressed in cm^2^ and keV/μm, respectively. This result is equivalent to the statement that the destruction cross section and the sputtering yield of valine are approximately proportional to the deposited dose in the material.

In the data shown in Figure 13, one extra cross section is the value corresponding to valine irradiated by 90 MeV ^127^I^14+^ projectiles. This result was obtained by Salehpour et al. [23], using low flux projectiles (1000 ions/s) and acquiring the time-of-flight spectrum of the desorbed ions. The electronic stopping power of 90 MeV ^127^I^+14^ ions in valine is 1.04 × 10^4^ keV/μm. Besides the desorption valine products, the ejected ions were composed by protonated valine (M + H)^+^ and by the cluster ion series (M_n_ + H)^+^, where *n* runs up to ~20. During the irradiation with up to 2 × 10^11^ projectiles, the yield of emitted cluster ions is exponentially quenched. The damage (destruction) cross sections of the protonated (M + H)^+^ and deprotonated (M-H)^−^ species were determined to be 68 (±18) × 10^−14^ cm^2^ and 44 (±15) × 10^−14^ cm^2^, respectively. We note that this is a direct sputtering measurement of charged particles. Although neutral particles are dominant in the induced desorption, the measured cross section corresponds to *radiolysis*, that is, only to the destruction cross section. Indeed, the sputtering yield (of ion species) reflects the evolution of the valine concentration on the sample surface which is ruled by its radiolysis. Therefore, the radiolysis cross section for the 90 MeV ^127^I^14+^ beam shown in Figure 13 appears below the line which represent values produced by both sputtering and radiolysis. 

### 3.2. Astrophysical Implications

**Figure 14:**
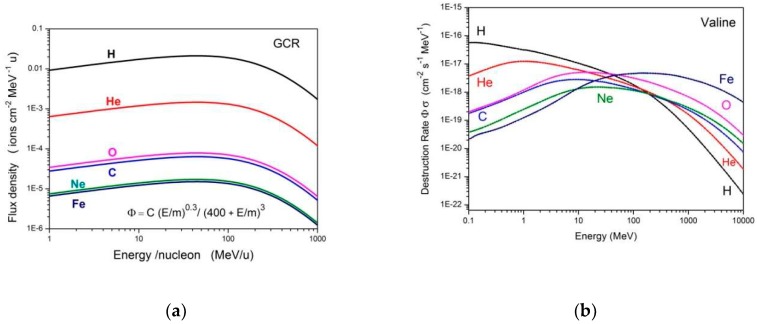
(**a**) Galactic cosmic ray (GCR) flux densities as predicted by Shen et al. [37]. (**b**) Valine destruction rate dependence on cosmic ray energy.

The assumption that Equation (1) holds for the destruction of solid valine by all cosmic rays regardless of their energy allows evaluating its absolute destruction rate. For each cosmic ray constituent, j, the corresponding partial destruction rate is:

(2) $$R_{j}=\;\overset{\infty }{\underset{0}{\int }}\frac{d\Phi_{j}}{dE}\left(E\right)\sigma_{d,j}^{ap}\left(E\right)dE$$

where *E* is the kinetic energy of species *j*, *Φ_j_*(*E*) is its flux density and *σ_d_,_j_*(*E*) its apparent destruction cross section given by Equation (1); for the current calculations, we have considered a = 1.0 × 10^−20^ cm^3^/keV and n = 1.0. Shen et al. [37] proposed a model in which the galactic cosmic ray (GCR) flux densities present the analytical expression:

(3) $$\frac{d\Phi_{j}}{dE}\left(E\right)=C_{j}\frac{E^{0.3}}{{\left(E+E_{0}\right)}^{3}}$$

where *E* is the GCR energy per nucleon, *E*_0_ is a parameter considered here as equal to 400 MeV, and *C_j_* is determined from the GCR abundances. For the current calculations, we considered abundances and fluxes presented in Table 1 and Figure 2 of ref. Shen et al. [37]; the fluxes are multiplied by 2π sr, for taking into account the isotropic GCR incidence on a spot located in a flat surface. The flux density dependence on E/m (Equation (3)) is presented in Figure 14a and the destruction rate dependence on E/m (Equation (2)) is shown in Figure 14b. The dominant effect of H and He ions at low GCR energy (and of Fe ions at high energy) is evident. The integral in Equation (2) was performed from 10 keV up to 10 GeV/u. Table 5 presents the C_j_ values (integrated over 2π sr) used, the R_j_ results for j = H. He, C, O, Ne and Fe, as well as the respective partial half-lives, defined as ln(2)/R_j_, the total destruction rate and total half-life.

The valine radiolysis and sputtering by GCR are ruled by their iron constituents. The half-life of valine in the ISM (InterStellar Medium) is estimated to be about one million years. Therefore, since no IR (infrared) signal of valine has been detected yet, we would like to encourage observations of valine rotational features by radio spectroscopy.

## 4. Materials and Methods

### 4.1. Ion Beam Irradiation of Valine

Irradiations using 1.5 MeV H^+^, He^+^, and N^+^ ion beams were performed at PUC-Rio and those with 230 MeV S^15+^ projectiles were carried out at GANIL. Pilling et al. [17] and Vignoli Muniz et al. [30] published detailed descriptions of the experimental set-ups, respectively. Chamber pressure was lower than 10^−6^ mbar. Samples were irradiated at room temperature (~300 K) and effects for lower temperature (40–300 K) on shape and integrated absorbance of valine vibrational bands were reported recently by da Costa and da Silveira [33].

Samples were prepared as follows: d-Valine (99.5% of purity, delivered by Sigma Aldrich, São Paulo, Brazil) was dissolved in a solution of water (40% *v*/*v*) and ethanol (60% *v*/*v*). The mixture was shaken in ultrasound machine up to a complete and homogeneous solution has been obtained. For the experiments at the Van de Graaff Laboratory, this solution was sprayed onto a ZnSe window, a 2 mm thick and 13 mm diameter disk. For the GANIL experiment, drops of this solution were deposited onto the ZnSe window. Substrate and solution were heated at 100 °C for enhancing the solvent evaporation. Sample thicknesses, T_k_, were determined from T_k_ = S ln(10) M/ρ N_A_ A_ν_**,** where S is the integrated absorbance of a given band, A_ν_ is its A-value (band strength), ln(10) = 2.303, M = 117.15 g/mol the valine molecular weight, ρ = 1.32 g/cm^3^ the valine mass density and N_A_ the Avogadro number. Two bands have been used for this calculation: the one at 948 cm^−1^ with A_ν_ = 1.04 × 10^−18^ cm/molec, and the one at 716 cm^−1^ with A_ν_ = 3.37 × 10^−18^ cm/molec. The results agree within 50% for several samples.

At the Van de Graaff Laboratory, the samples were kept inside the analytical chamber on a stainless steel sample-holder at room temperature (pressure below 10^−6^ mbar). Ion beam fluence was determined from current measurements via two nanoampere meters connected to a beam collimator and to a Faraday cup, respectively; both electrodes were biased to +90 V, for minimizing secondary electron emission. Infrared spectra were acquired in transmission mode by a JASCO FTIR-4100 spectrometer. The 4000–600 cm^−1^spectral region was inspected by averaging 70 scans with 2.0 cm^−1^ resolution. Absorbances were measured by integrating areas after subtracting a linear background defined within the band limits. At GANIL, the Casimir chamber was installed at the SME irradiation beamline. This is a dedicated beamline for sample irradiation with homogeneous irradiation field (*xy*-scanning of the ion beam) and permanent control of the beam flux, which was about 1 × 10^9^ cm^−2^·s^−1^ for MeV S^15+^ projectiles^.^ The residual pressure was about 2 × 10^−6^ mbar; FTIR acquisition was done by a Nicolet Magna 550 spectrometer over the 4000–600 cm^−1^ range with 1-cm^−1^ resolution.

The observed CO_2_ bands are produced by molecules outside the chamber and do not interfere with the valine radiolysis. The ion beam electronic (S_e_) and nuclear (S_n_) stopping powers for valine were determined through the software SRIM [38]; S_e_ >> S_n_ for all used ion beams, ion–atom collisions occurred in the electronic energy loss regime.

### 4.2. Cross Sections from Modelling of Fluence Dependent Absorption

Determination of molecular destruction and formation cross sections requires monitoring abundance rates of molecular species. This can be done by infrared spectroscopy, using the Beer–Lambert law, N = ln10 S/A*_ν_*, which connects the integrated absorbance S with the molecular column density N for each substance in the sample—provided that the A-value parameter, A*_ν_*, is known. A major concern is that this parameter varies with the chemical environment of the molecule, which in turn depends on temperature, solid state phase (crystallization/amorphization) and relative abundance of molecular constituents of the sample, quantities that change with irradiation.

Common models describing the dependence of integrated absorbance on projectile fluence consider that S(F) variation is due to sputtering (ejection of valine or fragments), radiolysis (chemical reactions) and—sometimes—by beam induced phase transition (molecular rearrangement in the sample, such as crystallization, amorphization, and compaction). Since molecular rearrangement processes are accomplished faster than the sample destruction, data analysis may be separated in two parts: a first one, when absorption changes by the rearrangement and by other processes, and a second one, when only sputtering and radiolysis proceed. Since the compaction process does not alter the column density N of a molecular film (although its thickness may vary), absorbance changes due to A-value variations are considered in the current model to avoid misinterpretation of destruction cross sections. Inside the material (where there is no sputtering), the decrease rate of the precursor concentration is proportional to the concentration itself and, therefore, this concentration decreases as exp(−σ_d_ F) with fluence. Accordingly, at the material surface, the precursor sputtering yield, Y, also decreases exponentially for the same reason: the radiolysis dictates the reduction of the precursor concentration (Sundqvist et al. [25]).

Infrared spectroscopy in transmission mode measures the precursor column density as a function of fluence. Hereby, radiolysis and sputtering effects are simultaneously monitored:

(4) $$\frac{dN}{dF}=-\sigma_{d}N-Y$$

Considering the precursor sputtering yield Y = Y_0_ (C/C_0_) ≈ Y_0_ (N/N_0_), where Y_0_, C_0_ and N_0_ are the sputtering yield, the concentration at surface and the column density of the virgin sample. For fluences that are not too high, Equation (4) can be rewritten as:

(5) $$\frac{dN}{dF}\approx -\sigma_{d}N-Y_{0}\frac{N}{N_{0}}\equiv -\sigma_{d}^{ap}N$$

with the solution:

(6) $$N\left(F\right)\approx N_{0}\;\exp(-\sigma_{d}^{ap}F)$$

Equation (6) shows that the apparent destruction cross section $\sigma_{d}^{ap}=\;\sigma_{d}+\;Y_{0}/N_{0}$ is the quantity actually determined in FTIR measurements [29]. This experimental inability should not be regarded as a methodological deficiency. Rather, it reveals that the laboratorial simulation mimics correctly the combined effect of molecular dissociation and molecular sputtering that occurs when swift heavy ions impinge on a material surface in space.

In order to describe Equation (6) in terms of absorbance, the Beer–Lambert law is applied:

N(F) = ln10 S(F)/A*_v_*(F) and, N_0_ = ln10 S_p_/A*_v_*(0):

(7) $$\frac{S\left(F\right)}{S_{p}}=\frac{A_{v}\left(F\right)}{A_{v}\left(0\right)}\exp\left(-\sigma_{d}^{ap}F\right)$$

where *S_p_* is the “porous” initial absorbance, that is, the absorbance for the fresh sample as prepared. The ratio *A_v_*(0)/*A_v_*(*F*) describes compaction effects, and also explains why the different precursor bands do not evolve proportionally to each other as fluence increases. For compaction, it was found empirically that [39,40]:

(8) $$\frac{A_{v}\left(F\right)}{A_{v}\left(0\right)}=\;\frac{1-\zeta \;\exp\left(-\sigma_{c}F\right)}{1-\zeta }$$

where ζ = (S_0_–S_p_)/S_0_ is the relative porosity, understood as a (beam-dependent) parameter able to quantify the effect of porosity on the absorbance evolution of a given band. S_0_ is interpreted as being the initial integrated absorbance of a compacted sample; for non-compac ted materials, this value is obtained by extrapolating the integrated absorbance decay to F = 0, but using absorbances for an already compacted sample. Equation (8) takes into account the infrared spectrum change of a porous sample when it suffers compaction either by irradiation or by annealing (before irradiation); each band shows this absorbance change differently, reason why each band has its own ζ for a specific ion beam. The parameter σ_c_ is called compaction cross section; for σ_c_ F << 1, Equation (8) writes A*_v_*(F)/A*_v_*(0) ~ 1 + (ζ/1−ζ) σ_c_F, another way of seeing that absorbance may increase or decrease with fluence depending whether ζ is positive or negative, respectively.

Independently of compaction or other phase changes, A*_v_*(F) may also have a dependence on fluence due to the persistent increase of daughter molecules in the sample. Since concentrations vary exponentially, it is reasonable to assume in first order approximation that A*_v_*^j^(F) = A*_v_*^j^(0) exp(−Δσ_j_ F) for each vibrational band j. In this case, Equation (7) writes, excluding compaction:

(9) $$\frac{S_{j}\left(F\right)}{S_{p,j}}=\frac{A_{v}{}^{j}\left(F\right)}{A_{v}{}^{j}\left(0\right)}\exp\left(-\sigma_{d,j}^{ap}\;F\right)=\exp\left(-\left(\sigma_{d,j}^{ap}+\Delta \sigma_{j}\right)F\right)$$

The prediction is that absorbances decrease exponentially but not with the same σ_d_^ap^ for all bands; the dispersion is nevertheless small, once Δσ_j_ << σ_d,j_^ap^. Again, comparison among different systems should be made with caution, comparing cross sections for the same band or mean values.

## 5. Conclusions

Thin films of D-valine were irradiated by four MeV ion beams. Compaction, radiolysis and sputtering processes were analyzed by FTIR spectroscopy at room temperature. The main conclusions are:

1. Compaction effects are seen via absorption modification, which means band strength modification. This process is due to the destruction of pores and to phase changes in the solid sample.
2. The chemical effects of the irradiation by distinct ion beams on valine are similar except for their molecular destruction rates (or destruction cross sections), which vary according to the deposited dose.
3. The elimination of valine molecules from the irradiated sample proceeds by radiolysis and by sputtering. Radiolysis imposes that the sample column density decreases exponentially with beam fluence. Another consequence is that the precursor concentration on the sample surface varies also exponentially and, in turn, the precursor sputtering yield decreases accordingly.
4. Strong evidence that the sputtering yield decays exponentially during irradiation is given by mass spectrometry measurements. Indeed, Salehpour et al. [23] found this behavior for valine bombarded by ^127^I^14+^ ions. Furthermore, Ferreira-Rodrigues et al. reported similar results analyzing the sample surface by TOF-^252^Cf–PDMS for glycine radiolysis [22]. FTIR spectroscopy determines the loss rate of precursors: the technique cannot distinguish those sputtered from those dissociated. Accordingly, the apparent destruction cross section, σ_d_^ap^, is measured: it quantifies the combined effect of both processes. It is found that σ_d_^ap^ is approximately proportional to the electronic stopping power and, therefore, to the absorbed dose. This is an unexpected finding, since for condensed gases the literature indicates σ_d_^ap^ proportional to S_e_^n^, where *n* ~ 3/2 [28].
5. At the end of irradiation, valine was destroyed and their bands vanished. The still visible bands are due to valine’s daughter molecules. Structural attributions are attempted.
6. Band strengths evolve with fluence but not linearly; moreover, some A-values are more sensitive to fluence than others. This feature does not depend on the processing ion and is attributed to compaction and to the increase of product concentrations.
7. Concerning Astrophysics, using theoretical GCR flux distribution, the solid valine half-life is predicted to be about one million years. Recent work estimated solid adenine half-life to be 10 ± 8 million years (Vignoli Muniz et al. [30]). These results suggest that the search for valine by radio astronomy should be envisaged.
8. Concerning Astrobiology, 1.1 × 10^7^ Gy is the mean dose to destroy solid valine. This is a huge value for Biology standards. For human beings, 50 Gy is a typical lethal dose.
9. Concerning radiotherapy, the current results, obtained for valine, are actually typical for amino acids in general and even for biological material. Stopping powers are calculated for ion-atom interactions and biological materials are mostly formed by carbon, oxygen, nitrogen and hydrogen—the same atoms of the molecular structure of valine; biological material mass densities are close to 1 g/cm^3^, so that penetration depths are similar to valine. Therefore, the findings of this work may be a useful contribution for the understanding of microscopic processes in the damage of biological targets, and in particular, radiation protection and ion beam therapy.

## Figures and Tables

**Table 1:** Evolution of valine bands as the 1.5 MeV H^+^ and 230 MeV S^15+^ projectile fluences increase. Band identification and their band wavenumbers for valine before irradiation; wavenumber of products after H^+^ and S^15+^ irradiation. (a) split band at low temperature: da Costa and da Silveira [33]; (b) Kumar [35]; (c) Façanha Filho et al. (2008) [36]. Attributions are tentative. (*) strong absorbance; (**) low absorbance.

Vibration Mode	Band (cm^−1^)	Band Collapses into (Possible Species)	
		H^+^	S^15+^
NH_3_^+^ asy str	3150, 3050	-	-
CH_3_^+^ asy str	2960, 2940	2955, 2918 (C_2_H_6_ *)	2961, 2933 (C_2_H_6_ *)
CH_3_^+^ asy str	2880, 2850	2868, 2849 (C_2_H_6_ *)	2874 (C_2_H_6_ *)
N-H…O	2690, 2630, 2580	-	-
CH_3_ bend + NH_2_ rocking ^b^	~2109 (2153 + 2013) ^a^	-	-
CO_2_^−^ stretch ^c^	1640	-	-
NH_3_^+^ asy def.	1600, 1555	-	1595 (Amine)
-	-	1544, 1507	1516 (Nitro)
COO^+^ sy str	1520, 1430, 1390	1457	1469 (C_2_H_6_), 1394
-	-	-	1355 (Nitro)
COO^+^/CO	1340, 1320	1339	1329
CH_3_^+^ def.	1271, 948	-	1271
			885 (C_2_H_6_^**^)
CO_2_ Bend ^b^	775	-	
C-H out bend	716	716	

**Table 2:** Relative A-values (integrated absorbance ratios), before and during irradiation. Ratios refer to the 948 cm^−1^ band, taken as reference. F = 0 means non irradiated sample and F_ref_ is the fluence for which the absorbance ratios were measured (4th line). Sample thickness values are also shown.

	Ion Beam:	A-Value/A-Value (F_ref_)			
		H^+^ 1.5 MeV	He^+^ 1.5 MeV	N^+^ 1.5 MeV	S^15+^ 230 MeV
	Sample Thickness	2.4 μm	0.96 μm	0.23 μm	0.58 μm
Band (cm^−1^) (interval)	F_ref_ (ions cm^−2^)	5.8 × 10^14^	1.1 × 10^13^	4.39 × 10^12^	3.2 × 10^11^
2900* (3300–2400)	0	273	282	235	246
	F_ref_	768	602	270	303
1329 (1335–1301)	0	3.94	3.07	9.51	10.8
	F_ref_	5.23	6.34	10.5	13.8
1271 (1279–1261)	0	0.876	1.16	0.981	0.984
	F_ref_	0.955	1.70	0.997	1.22
948 (957–937)	reference	1	1	1	1
	reference	1	1	1	1
716* (726–705)	0	2.80	2.70	2.35	3.39
	F_ref_	5.05	3.31	2.54	3.85

**Table 3:** Band interval used for the integrated absorbance calculation. Apparent destruction cross sections obtained from individual fittings for 1.5 MeV, H^+^, He^+^ and N^+^, and 230 MeV S^15+^ ion irradiations. For S^15+^, Δσ_j_ is the discrepancy between the mean value and each individual σ_d_^ap^. (*) Overlapping with product’s band.

Band (cm^−1^)		Ion Beam					
		1.5 MeV				230 MeV	
Band Interval	Band Maximum	H^+^	He^+^	N^+^	S^15+^		
		σ_d_^ap^ (10^−14^ cm²)				Δσ_j =_ (σ_d_^ap^)_mean_ − (σ_d_^ap^)_j_ (10^−14^ cm^2^)	Observations
3300–2400	2900*	14	3.3	7.2	29	4.7	no compaction, tholins at the end
1335–1304	1329	16	3.8	7.6	30	3.7	no compaction
1279–1261	1271	33	4.9	11	39	−5.3	disappears at the end
957–937	948	27	7.8	13	37	−3.3	
782–763	775	-	4.0	-	34	-0.3	σ_c_(S) = 50 × 10^−14^ cm^2^σ_c_(He) = 82 × 10^−14^ cm^2^
726–705	716 *	42	7.2	15	33	0.7	no compaction, tholins peaks too small
Mean value	σ_d,j_^ap^ ± Δσ_j_	26 ± 16	5.4 ± 2.1	11 ± 4	33.7 ± 4	0	12% rms error

**Table 4:** Relevant characteristics of the four irradiations: beam energy, electronic and nuclear stopping powers for valine, initial absorbances for the 716 and 948 cm^−1^ bands, initial column density, initial sample thickness, maximum beam penetration (range) and measured apparent destruction cross section.

Ion Beam	H^+^	He^+^	N^+^	S^15+^
Energy (MeV)	1.5	1.5	1.5	230
S_e_ (keV µm^−1^)	26.7	252	998	1690
S_n_ (keV µm^−1^)	0.0187	0.256	7.84	1.03
S_p_ 716 cm^−1^ (cm^−1^)	1.82	0.903	0.20	0.688
S_p_ 948 cm^−1^ (cm^−1^)	0.173	0.345	0.0849	0.151
**Valine sample**				
N_0_ (10^17^ molec/cm^2^)	8.12	6.56	1.61	4.00
Tk (µm)	2.4	0.96	0.23	0.58
Range (µm)	35	5.8	2.4	97
*σ_d_*^ap^ (10^−14^ cm^2^)	26 ± 16	5.4 ± 2.1	11 ± 4	34 ± 4

**Table 5:** The C_j_ flux parameter, the destruction rate R_j_ (in second^−1^ and in million-year^−1^), and the half-live (in million-year) for each GCR species j.

j	C_j_ Ions cm^−2^ s^−1^ MeV^−1.7^	R_j_ (10^−16^ s^−1^)	R_j_ (Ma^−1^)	τ_1/2_ = ln(2)/R_j_ (Ma)
**H**	5.96 × 10^5^	7.1	0.022	31
**He**	4.11 × 10^4^	7.5	0.024	29
**C**	1.79 × 10^3^	8.8	0.028	25
**O**	2.22 × 10^3^	28	0.088	7.9
**Ne**	438	12	0.038	18
**Fe**	425	170	0.54	1.2
**Total**	6.42 × 10^5^	230	0.74	0.94

## References

[1] Marzzoco A., Torres B.B. (2015). Bioquímica Básica.

[2] Pizzarello S., Feng X., Epstein S., Cronin J.R. (1994). Isotopic analyses of nitrogenous compounds from the Murchison meteorite: Ammonia, amines, amino acids, and polar hydrocarbons. Geochim. Cosmochim. Acta.

[3] Botta O., Bada J.L. (2002). Extraterrestrial Organic Compounds in Meteorites. Surv. Geophys..

[4] Elsila J.E., Glavin D.P., Dworkin J.P. (2009). Cometary glycine detected in samples returned by Stardust. Meteorit. Planet. Sci..

[5] Kuan Y., Charnley S.B., Huang H., Tseng W., Kisiel Z. (2003). Interstellar Glycine. Astrophys. J..

[6] Snyder L.E., Lovas F.J., Hollis J.M., Friedel D.N., Jewell P.R., Remijan A., Ilyushin V.V., Alekseev E.A., Dyubko S.F. (2005). A Rigorous Attempt to Verify Interstellar Glycine. Astrophys. J..

[7] Cunningham M.R., Jones P.A., Godfrey P.D., Cragg D.M., Bains I., Burton M.G., Calisse P., Crighton N.H.M., Curran S.J., Davis T.M. (2007). A search for propylene oxide and glycine in Sagittarius B2 (LMH) and Orion. Mon. Not. R. Astron. Soc..

[8] Jones P.A., Cunningham M.R., Godfrey P.D., Cragg D.M. (2007). A Search for biomolecules in Sagittarius B2 (LMH) with the Australia Telescope Compact Array. Mon. Not. R. Astron. Soc..

[9] Abramov O., Mojzsis S.J. (2009). Microbial habitability of the Hadean Earth during the late heavy bombardment. Microb. Habitability Hadean Earth Late Heavy Bombard..

[10] Chyba C., Sagan C. (1992). Endogenous production, exogenous delivery and impact-shock synthesis of organic molecules: An inventory for the origins of life. Nature.

[11] Rothard H., Domaracka A., Boduch P., Palumbo M.E., Strazzulla G., da Silveira E.F., Dartois E. (2017). Modification of ices by cosmic rays and solar wind. J. Phys. B At. Mol. Opt. Phys..

[12] Diehl J.F. (2002). Food irradiation—Past, present and future. Radiat. Phys. Chem..

[13] Silindir M., Özer Y. (2012). The Effect of Radiation on a Variety of Pharmaceuticals and Materials Containing Polymers. PDA J. Pharm. Sci. Technol..

[14] Adaligil E., Patil K., Rodenstein M., Kumar K. (2019). Discovery of Peptide Antibiotics Composed of d-Amino Acids. ACS Chem. Biol..

[15] Lam H., Oh D.-C., Cava F., Takacs C.N., Clardy J., de Pedro M.A., Waldor M.K. (2009). D-Amino Acids Govern Stationary Phase Cell Wall Remodeling in Bacteria. Science.

[16] Gerakines P.A., Hudson R.L., Moore M.H., Bell J.-L. (2012). In situ measurements of the radiation stability of amino acids at 15–140 K. Icarus.

[17] Pilling S., Mendes L.A.V., Bordalo V., Guaman C.F.M., Ponciano C.R., da Silveira E.F. (2013). The Influence of Crystallinity Degree on the Glycine Decomposition Induced by 1 MeV Proton Bombardment in Space Analog Conditions. Astrobiology.

[18] Pilling S., Nair B.G., Escobar A., Fraser H., Mason N. (2014). The temperature effect on the glycine decomposition induced by 2 keV electron bombardment in space analog conditions. Eur. Phys. J. D.

[19] Maté B., Tanarro I., Escribano R., Moreno M.A., Herrero V.J. (2015). Stability of extraterrestrial glycine under energetic particle radiation estimated from 2 kev electron bombardment experiments. Astrophys. J..

[20] Souza-Corrêa J.A., da Costa C.A.P., da Silveira E.F. (2019). Compaction and Destruction Cross-Sections for α-Glycine from Radiolysis Process via 1.0 keV Electron Beam as a Function of Temperature. Astrobiology.

[21] Peeters Z., Botta O., Charnley S.B., Ruiterkamp R., Ehrenfreund P. (2003). The Astrobiology of Nucleobases. Astrophys. J..

[22] Ferreira-Rodrigues A.M., Homem M.G.P., Naves de Brito A., Ponciano C.R., da Silveira E.F. (2011). Photostability of amino acids to Lyman α radiation: Glycine. Int. J. Mass Spectrom..

[23] Salehpour M., Håkasson P., Sundqvist B. (1984). Damage cross sections for fast heavy ion induced desorption of biomolecules. Nucl. Instrum. Methods Phys. Res. Sect. B Beam Interact. Mater. At..

[24] Becker O., Della-Negra S., Le Beyec Y., Wien K. (1986). MeV Heavy Ion induced desorption from insulating films as function of projectile velocity. Nucl. Instrum. Methods Phys. Res..

[25] Sundqvist B., Hedin A., Håkasson P., Salehpour M., Säve G. (1986). Sputtering of Biomolecules by fast heavy ions. Nucl. Instrum. Methods Phys. Res. Sect. B Beam Interact. Mater. At..

[26] De Barros A.L.F., Domaracka A., Andrade D.P.P., Boduch P., Rothard H., da Silveira E.F. (2011). Radiolysis of frozen methanol by heavy cosmic ray and energetic solar particle analogues: Radiolysis of frozen methanol by heavy cosmic ray and energetic solar particle analogues. Mon. Not. R. Astron. Soc..

[27] Godard M., Féraud G., Chabot M., Carpentier Y., Pino T., Brunetto R., Duprat J., Engrand C., Bréchignac P., d’Hendecourt L. (2011). Ion irradiation of carbonaceous interstellar analogues: Effects of cosmic rays on the 3.4 *μ* m interstellar absorption band. A&A.

[28] Andrade D.P.P., de Barros A.L.F., Pilling S., Domaracka A., Rothard H., Boduch P., da Silveira E.F. (2013). Chemical reactions induced in frozen formic acid by heavy ion cosmic rays. Mon. Not. R. Astron. Soc..

[29] Mejía C.F., de Barros A.L.F., Bordalo V., da Silveira E.F., Boduch P., Domaracka A., Rothard H. (2013). Cosmic ray–ice interaction studied by radiolysis of 15 K methane ice with MeV O, Fe and Zn ions. Mon. Not. R. Astron. Soc..

[30] Vignoli Muniz G.S., Mejía C.F., Martinez R., Augé B., Rothard H., Domaracka A., Boduch P. (2017). Radioresistance of Adenine to Cosmic Rays. Astrobiology.

[31] Weast R.C. (1981). CRC Handbook of Chemistry and Physics.

[32] Ada Bibang P.C.J., Aditya N.A., Augé B., Boduch P., Desfrançois C., Domaracka A., Lecomte F., Manil B., Martinez R., Muniz G.S.V. (2019). Ion radiation in icy space environments: Synthesis and Radioresistance of Complex Organic Molecules. Low Temp. Phys..

[33] Da Costa C.A.P., da Silveira E.F. (2019). Valine infrared absorbance at cryogenic temperatures. Low Temp. Phys..

[34] Sagan C., Khare B.N. (1979). Tholins: Organic chemistry of interstellar grains and gas. Nature.

[35] Kumar S. (2011). Spectroscopic studies of valine and leucine molecules a comparative study. Vib. Spectrosc..

[36] Façanha Filho P.F., Freire P.T.C., Lima K.C.V., Mendes Filho J., Melo F.E.A., Pizani P.S. (2008). High temperature Raman spectra of L-leucine crystals. Braz. J. Phys..

[37] Shen C.J., Greenberg J.M., Schutte W.A., van Dishoeck E.F. (2004). Cosmic ray induced explosive chemical desorption in dense clouds. A&A.

[38] Ziegler J.F., Ziegler M.D., Biersack J.P. Interactions of Ions with Matter. srim.org.

[39] Mejía C., de Barros A.L.F., Seperuelo Duarte E., da Silveira E.F., Dartois E., Domaracka A., Rothard H., Boduch P. (2015). Compaction of porous ices rich in water by swift heavy ions. Icarus.

[40] De Barros A.L.F., da Silveira E.F., Fulvio D., Rothard H., Boduch P. (2016). Ion irradiation of ethane and water mixture ice at 15 k: Implications for the solar system and the ism. Astrophys. J..

